# Metalinguistic and Reading Skills in a Sample of Colombian Children with Attention Deficit Hyperactivity Disorder

**DOI:** 10.3390/children11111309

**Published:** 2024-10-29

**Authors:** Diana Montoya-Londoño, Daniel Landínez-Martínez, Lorena Aguirre-Aldana, Carmen Dussán-Lubert, Antonio Partida-Gutierrez de Blume

**Affiliations:** 1 Educational Studies Department, Faculty of Arts and Humanities, Universidad de Caldas, Manizales 170001, Colombia; diana.montoya@ucaldas.edu.co; 2 Faculty of Social and Human Sciences, Universidad de Manizales, Manizales 170001, Colombia; laguirrea@umanizales.edu.co; 3 Faculty of Health Sciences, Universidad de Manizales, Manizales 170001, Colombia; 4 Social Sciences, Health and Welfare Faculty, Luis Amigo Catholic University, Manizales 170001, Colombia; 5 Department of Mathematics, Faculty of Exact and Natural Sciences, Universidad de Caldas, Manizales 170001, Colombia; carmen.dussan@ucaldas.edu.co; 6 Georgia Southern University, Statesboro, GA 30458, USA; agutierrez@georgiasouthern.edu

**Keywords:** metalinguistic skills, reading accuracy, reading comprehension, reading speed, attention deficit hyperactivity disorder, children’s neuropsychological assessment

## Abstract

**Objective:** This study aimed to examine metalinguistic skills and reading processes in children diagnosed with ADHD, compared to a matched control group. **Method:** An explanatory experimental design was employed, involving a sample of 194 children from Manizales, comprising 97 children diagnosed with ADHD and 97 controls. The study utilized tasks from the Children’s Neuropsychological Assessment (CNA) protocol to assess metalinguistic and reading abilities. **Results:** Children with ADHD exhibited significantly lower performance across all metalinguistic and reading tasks compared to the control group, except for spelling and silent reading comprehension tasks. **Conclusions:** These findings corroborate previous research conducted in Manizales, underscoring the specific challenges in metalinguistic and reading domains among children with ADHD. Future research should explore the influence of working memory on phonological awareness and its implications for metalinguistic skills and reading development.

## 1. Introduction

Attention Deficit Hyperactivity Disorder (ADHD) is recognized as the most prevalent neurodevelopmental disorder in childhood, often compromising quality of life, comprehensive human development, and academic performance in affected children. Despite being frequently diagnosed early in life, there is considerable controversy surrounding its prevalence, with estimates ranging from 11.3% to 17% in the United States [1], 17.1% in Colombia [2], and a more recent meta-analysis estimating a global prevalence of approximately 7.2% among children and adolescents [3].

Research on ADHD has extensively explored the disorder’s heterogeneity, investigating its behavioral manifestations, etiology, developmental trajectories, comorbidities, and responses to various interventions. The disorder has three primary subtypes—predominantly inattentive, predominantly hyperactive-impulsive, and combined—varied clinical presentations, complicating diagnosis and treatment. In addition to these subtypes, ADHD is frequently associated with comorbidities such as anxiety, depression, and learning disabilities, which exacerbate its impact on academic, social, and occupational functioning. These functional impairments lead to significant psychosocial consequences, including a diminished quality of life and increased familial stress. While ADHD often persists into adulthood, the trajectory of the disorder varies, influenced by early intervention, comorbid conditions, and environmental factors [4,5]. However, the academic and emotional dimensions associated with ADHD have received comparatively less attention, despite substantial evidence documenting the impact of neuropsychological and behavioral profiles on academic outcomes [6]. Cognitive prerequisites for academic skills, particularly neuropsychological predictors of reading, have been identified as critical factors in understanding these academic challenges [7,8].

Some studies have highlighted that children with ADHD often exhibit a neuropsychological profile characterized by executive dysfunction. These may include difficulties with goal setting, behavioral initiation, inhibition, verbal fluency, temporal organization, and tasks involving classification and categorization, all of which are associated with frontal lobe functioning [9]. Consequently, children diagnosed with ADHD may also struggle with metalinguistic skills and the phonological components of language, which are essential for the acquisition and development of reading and writing skills [10].

Moreover, research indicates that while specific learning disorders can occur independently of behavioral issues, the presence of behavioral disorders, such as those seen in ADHD, frequently align with academic challenges. The significant comorbidity between ADHD and poor academic performance, particularly in tasks requiring sustained attention, suggests that children with ADHD often demonstrate lower academic achievement and weaker adherence to academic responsibilities [11,12]. Additionally, difficulties in reading and mathematical skills observed in children with ADHD may be linked to neuropsychological impairments, particularly within the inattentive dimension, which may stem from genetic and phenotypic factors intrinsic to the disorder [13].

The neuropsychological profile of children with ADHD, particularly in the context of reading comprehension, seems to be closely associated with deficits in metalinguistic skills (Table 1). Several studies have explored the relationship between various personal and social factors and reading comprehension, highlighting the role of personal-cognitive factors such as phonological processing, working memory, self-monitoring, and inference-making, as well as social factors like goal setting, motivation, expectations, and prior knowledge [14].

In terms of personal cognitive factors, phonological awareness—a metalinguistic skill—has been identified as a key predictor of reading development. This skill enables the recognition of the internal structure of words, facilitating the identification and manipulation of phonemes and syllables, which are critical in the decoding process that underpins reading proficiency [15].

**Table 1:** Major research findings that have examined the relationship between metalinguistic skills and reading abilities in children with ADHD.

Method	Country	Findings	Study
Sample: Children aged 8 to 11 years diagnosed with ADHD Aim: To describe and compare the neuropsychological performance of children diagnosed with ADHD relative to that of a control group.	Colombia	Children in the inattentive ADHD group demonstrated significantly lower levels of semantic fluency compared to the control group. However, no statistically significant differences were observed between the groups regarding performance on the phonological fluency task.	[16]
Sample: Children aged 6 to 11 years diagnosed with ADHD. Aim: To compare the behavioral and neuropsychological skills of children with ADHD to those of a control group.	Colombia	No significant differences were identified between the ADHD and control groups in the evaluation of verbal comprehension processes and vocabulary level. However, children in the inattentive ADHD group demonstrated significantly lower performance in verbal fluency tasks compared to the control group, indicating specific deficits in language-related executive functions.	[17]
Sample: Children with an average age of 8 years and from a high socioeconomic background Aim: To determine the impact of ADHD on logical reasoning and metapragmatic knowledge	Chile	Children with ADHD demonstrated significantly lower levels of cognitive and linguistic skill development—specifically, verbal analogical reasoning and metapragmatic knowledge—compared to the healthy control group.	[18]
Sample: Children aged 9 to 13 years old Aim: To Compare the performance in cognitive-linguistic and reading skills between students with ADHD and students without a diagnosis of potential specific learning or behavioral disorders.	Brazil	Students in the control group outperformed children with ADHD on tasks involving phonetic and syllabic manipulation and addition, which were not considered to be associated with a phonological language disorder.	[19]
Sample: Children aged 12 to 16 years old Aim: Establish the potential specificity of deficits in linguistic and executive functioning in students with ADHD and reading comprehension difficulties (RCD), and identify the deficit profile of the comorbid group (ADHD + RCD) compared to a control group.	España	The ADHD + RCD group exhibited the most significant linguistic deficits, followed by the RCD group. Greater linguistic and executive deficits were observed in the RCD group and the ADHD group. Finally, the comorbid group (ADHD + RCD) experienced deficits in both linguistic and executive skills.	[20]
Sample: Children aged 6 to 14 years old Aim: To compare the neuropsychological performance in language processing between children with ADHD and a control group, and to establish correlations between their performance on academic and neuropsychological tasks	Colombia	Significant differences were established between the mean scores of metalinguistic skills for the sound counting task between the I-ADHD group and the control group. A significant correlation was observed between performance on academic and neuropsychological tasks from the Neuropsychological Assessment Battery (ENI) used to evaluate language. A positive correlation was found between the performance of children with ADHD in reading and all language tasks employed in the evaluation.	[21]
Sample: Children aged 8 to 12 years with ADHD. Aim: Characterize and compare the performance of students with ADHD in metalinguistic and reading skills relative to the performance of children with high proficiency in both evaluated skills.	Brazil	The performance of children with ADHD was lower in reading skills and non-word repetition tasks compared to children in the control group. Differences were observed between the groups regarding initial and final phoneme identification, as well as in the addition and subtraction of syllables and phonemes, and in phonetic segmentation. No statistically significant differences were found in reading comprehension between the groups.	[22]
Sample: Children aged 7 to 11 years old with and without ADHD Aim: Describe the presence of specific learning disorders (SLD) in reading among a group of children from the city of Manizales diagnosed with ADHD.	Colombia	The most significant difference between cases and controls is observed in the task of reading a text aloud, with children with ADHD scoring lower compared to the control group	[23]
Sample: Children aged 8 to 12 with a diagnosis of ADHD and a control group. Aim: To determine the effect of language comprehension and executive functions on reading comprehension in children diagnosed with ADHD.	Taiwan	The ADHD group exhibited lower scores in language and reading comprehension compared to the control group. Both groups showed equivalent scores on the Tower of London task (planning), but differed in the go/no-go tasks (inhibitory control), Wisconsin Card Sorting Test (WCST) (flexibility), and two tasks of verbal and spatial working memory (SWM and WM). Inhibition and verbal WM were significant predictors of reading comprehension in the ADHD group.	[24]
Sample: Children aged 8 to 12 with a diagnosis of ADHD and a control group. Aim: To compare language performance of children with ADHD to two samples: children with language disorder and Children with neurotypical development (controls).	Greece	Children with ADHD performed worse than their peers in the control group, but better than the group with language disorder. In pragmatics, children with ADHD had numerically lower performance than any other group, and they also showed difficulties with linguistic skills, particularly with structural language.	[25]

Several studies suggest that children diagnosed with ADHD typically exhibit significantly lower levels of cognitive and linguistic development—particularly in areas such as verbal analogical reasoning and metapragmatic knowledge—compared to control groups. These children also tend to display more pronounced linguistic deficits.

When comparing cases to controls, findings indicate that children with ADHD, regardless of subtype, generally score lower on tasks involving the initial and final identification of phonemes, the addition and subtraction of syllables and phonemes, as well as various phonetic segmentation processes. Moreover, children with ADHD also perform worse than controls in reading text aloud.

Among ADHD subtypes, the inattentive subtype appears to experience the most severe impairments, with lower scores in reading-related tasks, particularly in non-word repetition, semantic verbal fluency, and metalinguistic skills, especially sound counting tasks. In contrast, the control group demonstrated superior performance in tasks involving phonetic and syllabic manipulation and addition.

No significant differences were found between ADHD subtypes and controls in vocabulary, phonological fluency, verbal comprehension, or overall reading comprehension. However, a positive correlation was observed between all reading and language tasks evaluated.

In the context of the literature review, there appears to be a growing trend in researching the characteristics of metalinguistic skills and reading processes in samples of children diagnosed with ADHD compared to control samples, particularly in Spanish-speaking countries. This contrasts with studies involving other language groups, although there are also studies in countries where languages such as Chinese and Greek are spoken.

This phenomenon may be attributed to the fact that, from the early years of learning Spanish, children encounter more grammatical difficulties in this language than in most other languages. For instance, while some languages do not employ gender in nouns or feature irregular verbs that closely resemble their infinitives, Spanish has different grammatical rules that can reveal language difficulties early in development. These include aspects such as the use of the subjunctive mood and the complexity of verb conjugations, given that verbs change depending on the subject, unlike English, which only has an exception for the third person singular. Additionally, there are challenges related to accentuation, irony, double meanings, and the unique letters of the Spanish alphabet, such as “ñ” and “h” [26].

Specifically, it is observed that children with ADHD generally perform lower than control samples in metalinguistic skills and across most aspects of language and reading, which is associated with executive difficulties, particularly in working memory [27].

The study presented in this article aimed to establish the effect of the presence of ADHD on metalinguistic and reading skills in a sample of children from the city of Manizales diagnosed with Attention Deficit Hyperactivity Disorder compared to a control group. Additional goals of this study were to compare the neuropsychological performance in metalinguistic and reading skills of a sample of children with mixed and inattentive ADHD and a control group. Finally, to establish differences in neuropsychological performance in metalinguistic and reading skills between a sample of children with mixed and inattentive ADHD and a control group.

This study is of great interest given the high prevalence estimates of ADHD in Colombia, ranging from 15.86% to 17.1% [28,29], which is significantly higher than the global estimate of approximately 5% to 7% [27]. This elevated rate in Colombia may be explained by greater awareness of neurodevelopmental disorders in clinical and educational contexts; however, it may also reflect differences in the types of instruments used. Thus, there seems to be a need for greater international consensus to standardize measurement tests, allowing for more accurate comparisons between the prevalences of different populations and the multiple variations of individuals with ADHD [30].

## 2. Materials and Methods

### 2.1. Type of Research

The present study used an explanatory experimental design to rigorously investigate the cause-and-effect relationships between ADHD and metalinguistic and reading skills in children. This design allowed for a systematic exploration of both the differences between children with ADHD and a matched control group, as well as why these differences manifest. By manipulating the independent variable (the presence of ADHD) and controlling for confounding factors through careful participant matching, this study aimed to attribute any observed performance disparities directly to ADHD. The use of validated neuropsychological assessments further strengthened the reported findings, enabling robust conclusions to be drawn about the impact of ADHD on metalinguistic and reading processes (Table 2). Internal validity was ensured through initial equivalence by matching the two groups: cases and controls. This study was conducted according to the guidelines of the Declaration of Helsinki and approved by the Institutional Scientific Ethical Committee of the Universidad de Manizales, Colombia (ISET-03-22-0012); the date of approval by the ethics committee is 3 February 2022.

Based on the aim of this study, the following hypotheses were formulated:

**Null Hypothesis** **(H_0_).** *There is no significant difference in metalinguistic skills and reading processes between children diagnosed with ADHD and the control group.*

**Alternate Hypothesis** **(H_1_).** *Children diagnosed with ADHD exhibit significantly lower performance in metalinguistic skills and reading processes compared to the control group.*

### 2.2. Sample

The sample consisted of 194 school-aged children from Manizales. Half of the children had a diagnosis of ADHD. The case and control groups were matched based on sex, age, educational level, socioeconomic status, and type of educational institution. Each group (cases and controls) consisted of 24 girls and 73 boys, with ages ranging from 5 to 14 years (mean age: 9.4 years, standard deviation: 2.7 years), indicating a variability of 28.2% in this variable (participants in the study were homogeneous in terms of age). Regarding socioeconomic status, 19.6% of the evaluated children belonged to socioeconomic strata 1 and 2, 66.5% to strata 3 and 4, and 19.9% to strata 5 and 6. Similarly, 66.5% of the participants attended private schools, while the rest were enrolled in public schools. In the case group, 59.8% of the children were diagnosed with combined-type ADHD, and the remaining had predominantly inattentive ADHD. However, there is no significant difference between the inclusion criteria by sex (*p* value = 0.967), age (*p* value = 0.9798), educational level (*p* value = 0.819), and socioeconomic stratum (*p* value = 0.794). But for the type of educational institution, there is a difference (*p* value = 0.037), being greater in private schools the number of controls.

In this study, the diagnosis of ADHD in participating children was conducted by a multidisciplinary team, comprising licensed psychologists and psychiatrists, utilizing standardized diagnostic instruments, including clinical interviews and behavioral assessments in accordance with the DSM-5 criteria. This approach ensured a comprehensive and reliable diagnosis of the condition. Furthermore, the study adhered to rigorous ethical standards as approved by the Institutional Scientific Ethical Committee of the Universidad de Manizales. Prior to participation, written informed consent was obtained from parents or legal guardians, who were fully informed of the study’s objectives, procedures, and the voluntary nature of involvement, including the right to withdraw at any time without consequence. These steps were taken to ensure both the scientific validity and ethical integrity of the research.

### 2.3. Data Collection

#### Variables Used in the Research

The qualitative variables included in the research, along with their abbreviations and categories, are as follows:

- Inclusion Criterion: Case, Control.
- Sex: Male, Female.
- Socioeconomic Status (SES): Low, Medium, High.
- Type of Institution: Public, Private.
- ADHD Subtype: Combined, Inattentive.

Metalinguistic skills: Metalinguistic skills are a set of abilities that allow an individual to reflect on language. Specifically, tasks commonly included in the assessment of this construct evaluate phonemic synthesis (the ability to form words from phonemes), spelling (the ability to spell words), sound counting (the ability to count the sounds that make up words), and word counting (counting the words that comprise a sentence).

Reading ability is a complex process that encompasses two fundamental components: word recognition and reading comprehension. It is an academic skill that involves interpreting the meaning of a type of information (word, text) communicated through a language code. In particular, the assessment of this construct typically includes estimating accuracy, speed, and comprehension through tasks such as reading syllables, words, non-words, and sentences, as well as reading a text aloud and silently, and estimating speed per word in both modalities.

### 2.4. Procedure

A random selection was made from the list of schools provided by the Secretary of Education of Manizales, including 5 private and 15 public schools. The research team contacted the administrators of these institutions to present the research project. In schools that agreed to participate, a face-to-face meeting was held with the families of the children to present the study’s objectives and identify those who provided informed consent for participation.

Once the participant group was established, the team administered behavioral questionnaires and intellectual screening to determine the sample of cases and controls according to the study’s inclusion criteria.

Subsequently, three appointments were scheduled for assessment. First, a neuropsychiatric interview was conducted to rule out comorbidities, including depressive disorders, oppositional defiant disorder, conduct disorder, and specific learning disorders. Next, the neuropsychological evaluation protocol was applied, which included tasks for assessing metalinguistic and reading skills. This process required participants to attend two appointments of approximately 45 min each.

### 2.5. Inclusion Criteria

1. A minimum full-scale IQ of 85, based on the score from an abbreviated version of the Wechsler Scale, CX6 form [31].
2. A T-score of 65 or higher for the ADHD group, and a T-score of 50 or lower for the control group on the inattention and hyperactivity/impulsivity dimensions of the Conners questionnaires and checklists completed by parents and teachers [32,33,34,35].
3. Informed consent signed by parents or guardians.

### 2.6. Instruments

1. Screening to Determine Inclusion and Exclusion Criteria
2. Brief Questionnaire (Checklist) for ADHD Diagnosis from DSM-IV [32,33,35].
3. Conners’ Parent Rating Scale (CPRS) and Teacher Rating Scale (CTRS) [34].
4. WISC III [36], abbreviated form C6 x2: Vocabulary and Block Design subscales [31].
5. Semi-Structured Psychiatric Interview MINI-KID (Mini International Neuropsychiatric Interview for Children and Adolescents) (Table 3) [37].

### 2.7. Performance in Metalinguistic and Reading Skills

The protocol for evaluating metalinguistic skills and reading ability included various domains and subtests from the Children’s Neuropsychological Assessment (CNA) [38,39]. The goal of the CNA is to assess neuropsychological development in Spanish-speaking children aged 5 to 16 years. The evaluation encompasses 13 distinct cognitive domains: attention, constructive abilities, memory (including encoding and delayed recall), perception, oral language, metalinguistic skills, reading, writing, mathematics, visuospatial skills, conceptual abilities, and executive functions. In addition to these cognitive domains, the CNA also assesses manual laterality and examines the presence of soft neurological signs.

The specifications for the subtests used from the CNA Battery are as follows: For metalinguistic skills, tasks included phonetic synthesis, spelling, sound counting, and word counting. For reading measures, tasks included accuracy, comprehension, and reading speed [40,41]. Table 4 presents the tasks used for assessing metalinguistic skills from the CNA battery [41] applied in this research.

All instruments used in this study have been rigorously adapted and validated to ensure their cultural and contextual relevance for the Colombian population. This meticulous process ensured that the instruments were both culturally sensitive and scientifically robust for accurately assessing the variables of interest within the Colombian context.

### 2.8. Data Analysis

Based on the available data, a data matrix was constructed and subjected to the following statistical analysis using the Xlstat software, version 2014.

### 2.9. Variable Description

- Statistical Comparison of Means: Tasks of metalinguistic and reading skills were statistically compared between the case and control groups. For each comparison, Student’s t-test was used after validating the normality of the data through the Shapiro–Wilk test. Additionally, the homogeneity of variances was determined using Fisher’s F-test. If a variable did not show a normal distribution, the non-parametric Mann–Whitney U test was conducted [42].
- Correlation Analysis: Correlations between variables were analyzed separately for the case and control groups. Pearson’s correlation coefficient was used when the variables met the assumption of normality; otherwise, Spearman’s rank correlation coefficient was employed.

## 3. Results

### 3.1. Variable Description

For all the metalinguistic skills studied (Table 5), the control group showed higher mean scores and greater homogeneity compared to the case group. This suggests that the case group tends to be more heterogeneous in their performance on tasks such as phonological synthesis, sound counting, spelling, and word counting.

**Figure 1:**
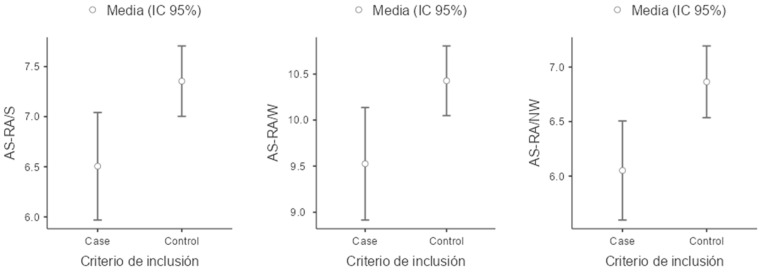
Boxplot of AS-RA/S (Academic Skills Reading Accuracy/Syllables), AS-RA/W (Academic Skills Reading Accuracy/words) and AS-RA/NW (Academic Skills Reading Accuracy/Non-Words).

**Figure 2:**
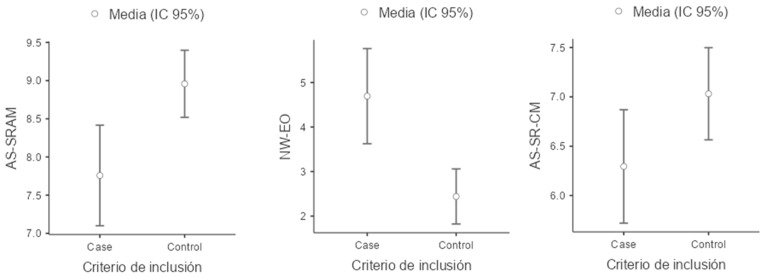
Boxplot of AS-SRAM (Academic Skills Sentence Reading Accuracy Measure), NW-EO (Number of Words with Errors in Oral Reading) and AS-SR-CM (Academic Skills Sentence Reading Comprehension Measure).

**Figure 3:**
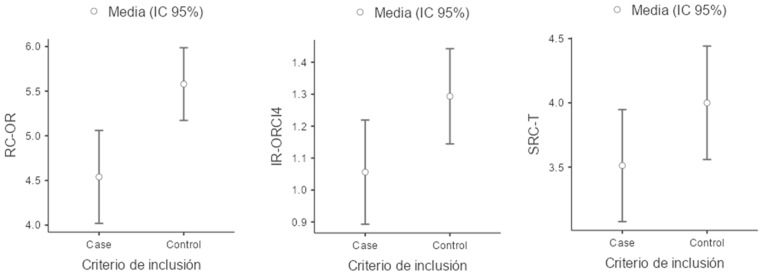
Boxplot of RC-OR (Reading Comprehension of Oral Reading), IR-ORCI4 (Inferential Response in Oral Reading Comprehension Raw score) and SRC-T (Silent Reading Comprehension of a Text).

**Figure 4:**
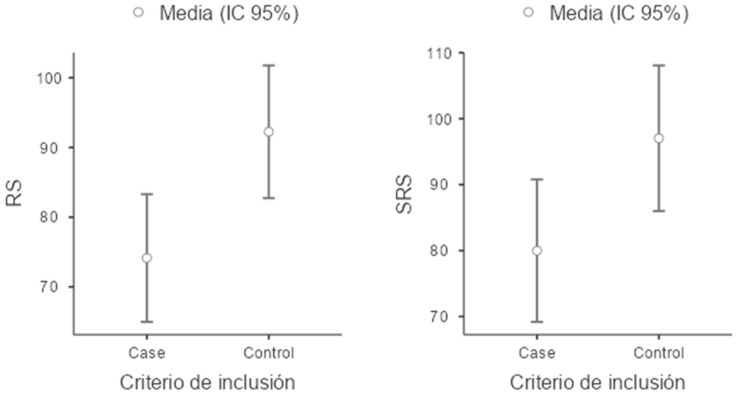
Boxplot of RS (Reading Speed) and SRS (Silent Reading Speed).

For the reading-related variables, children in the ADHD group scored lower on all evaluated measures compared to the control group. Notably, performance was particularly impaired in the measure of the number of errors in oral reading (NW–EO), where the ADHD group exhibited a higher mean number of errors than the control group. Additionally, a lower coefficient of variation was observed for this task, indicating that difficulties with oral reading errors are consistent and that the ADHD group is more homogeneous in the frequency of errors compared to the control group (Figure 1, Figure 2, Figure 3 and Figure 4).

### 3.2. Statistical Comparison of Metalinguistic Skills and Reading Tasks Between the Case and Control Groups

None of the variables related to metalinguistic skills and reading demonstrated a normal distribution (*p*-values were less than 0.032). Consequently, the Mann–Whitney U test was employed to compare cases and controls (Table 6 and Table 7).

Regarding metalinguistic skills, children in the case group exhibited lower mean scores compared to the control group in tasks involving synthesis, sound counting, and word counting. However, no statistically significant difference was observed between the groups in the spelling task (Table 6).

### 3.3. Comparison of Means for Reading-Related Variables

When comparing the means of variables related to reading, the children in the case group consistently exhibited lower mean scores than the control group in several areas, including: accuracy in reading skills with syllables, accuracy in reading skills with words, accuracy in reading skills with non-words, sentence accuracy in reading, the number of words read incorrectly during oral reading, oral reading comprehension, inferential response to oral reading comprehension (Item 4), oral reading speed, and silent reading speed. However, no significant differences were found in academic reading skills related to sentence comprehension and silent reading comprehension of a text (Table 7).

### 3.4. Correlation Analysis Between Variables for Each Group (Cases and Controls)

In the evaluation of metalinguistic skills for both the case and control groups, it was observed that an increase in scores for the synthesis task is positively correlated with higher scores in sound counting, spelling, and word counting tasks. Similarly, higher scores in sound counting are associated with increased scores in spelling and word counting tasks. Furthermore, children who achieve higher scores in spelling tend to obtain higher scores in the word counting task (Table 8).

The correlation matrix for reading-related variables in the case group (Table 9) reveals that these children exhibit a heightened sensitivity to the influence of performance in one task on their performance in other tasks. This sensitivity is evident across all reading skills, except for sentence precision (AS-SRAM) and non-word reading precision (AS-RA/NW).

For the variable “number of words with errors in oral reading” (NW-EO), all observed correlations were negative, indicating that as the number of words with errors increases, scores on the corresponding variables decrease.

In the control group, the tasks generally showed weaker correlations, with uncorrelated tasks highlighted in gray. Similarly, the NW-EO variable also demonstrated negative correlations.

## 4. Discussion

### 4.1. Sociodemographic Variables and the Presence of ADHD

In terms of sociodemographic variables, the children who participated in this study exhibited several notable characteristics. There was a higher representation of males compared to females, with 73 boys and 24 girls included in both the case and control groups. This finding aligns with previous research indicating a higher prevalence of ADHD in males, with an estimated ratio of 1:5, meaning that for every girl diagnosed with ADHD, there are at least five boys diagnosed with the disorder [43,44,45]. Epidemiological studies have consistently confirmed this gender disparity, reporting that ADHD is up to nine times more prevalent in boys than in girls in clinical samples [46,47,48].

The children in this study ranged in age from 5 to 14 years, with an average age of 9.4 years (SD = 2.7 years), coinciding with the typical age range for Colombian primary school education. During this educational stage, academic demands increase significantly compared to preschool, and children are expected to exhibit higher levels of self-regulation in their studies. As a result, attentional or behavioral difficulties become more evident and are less likely to be attributed to developmental immaturity, prompting families or schools to seek a clinical diagnosis of ADHD. This timing is consistent with recommendations from other studies suggesting that ADHD screening should be conducted between the ages of 7 and 8 years, corresponding to grades 2 and 3 of primary school, to detect ADHD and related developmental difficulties [49].

Previous studies have also identified the age range of 7.6 to 9 years as a critical period for ADHD diagnosis, as this is when parents and teachers are most likely to observe symptoms of hyperactivity, inattention, and/or behavioral problems that can affect classroom performance and overall academic achievement. These symptoms are often associated with poor academic performance, language and communication disorders, learning disorders, impulsive-aggressive behavior, and substance use risk, all of which are critical concerns for caregivers [17,45,50].

In the present study, the majority of diagnosed children belonged to socioeconomic strata 3 and 4 (66.4%), with lower percentages observed in strata 1 and 2 (19.6%) and strata 5 and 6 (19.9%). This finding is consistent with a previous study conducted in a central Colombian municipality, which found that the majority of children with ADHD were in the middle socioeconomic stratum (59.7%), with lower percentages in the lower (33.9%) and higher (6.3%) strata [28]. However, it contrasts with another study conducted in a Colombian capital city, which reported a higher prevalence of ADHD in the lower socioeconomic stratum (94%) among children aged 6 to 10 years in grades 1 through 4 of primary school [51].

These contrasting results may be explained by the influence of socioeconomic factors on cognitive and academic performance, such as the profession of the family head, the mother’s educational level, the primary source of family income, and living conditions. These factors have been associated with higher ADHD prevalence in lower socioeconomic strata (12,53). However, it is also possible that better clinical and educational conditions associated with middle and upper socioeconomic strata lead to earlier and more personalized diagnosis and treatment of ADHD. This is particularly relevant when these strata are associated with higher parental education levels, as ADHD diagnosis relies heavily on information provided by parents, family members, and teachers, and timely diagnosis appears to be related to the availability of objective informants who can provide relevant information about the clinical significance of symptoms and their impact on the child’s academic and family life [52].

In this study, 66.5% of the children attended private schools, with the remaining attending public schools. This finding is consistent with studies suggesting a higher prevalence of ADHD diagnoses among children from higher socioeconomic strata [53]. Additionally, in this study, 59% of the children in the case group were diagnosed with combined-type ADHD, while the remaining had predominantly inattentive-type ADHD. This finding is consistent with previous research reporting a higher prevalence of the combined subtype [44,54].

### 4.2. Metalinguistic Skills Profile in ADHD

Regarding metalinguistic skills, as shown in Table 5, the control group outperformed the case group and demonstrated greater homogeneity in their data. This result suggests that the case group is more heterogeneous in their performance on tasks involving synthesis, sound counting, spelling, and word counting.

Metalinguistic skills refer to an individual’s ability to distance themselves from the content of speech (discourse) to reflect on and manipulate the structure of language. These skills involve conscious reflection on various levels of the linguistic system [55,56]. According to some authors, metalinguistic skills are related to the formal aspects of language: phonological, morphological, syntactic, and lexical [57]. These skills typically develop around the age of 5 or 6 and gradually increase throughout the school years, playing a crucial role in successful reading acquisition [58,59,60].

The lower performance in metalinguistic skills observed in the case group compared to the control group is consistent with findings from three studies conducted in Brazil. The first study, conducted with 9-year-old children, aimed to describe reading skills in children with ADHD. The study found that the evaluated children performed below expectations on tasks related to lexical, syntactic, and semantic processes [9]. The second study, conducted with children aged 9 to 13 years, aimed to compare cognitive-linguistic and reading skills in children with ADHD (Group 1) and children without learning or behavioral disorders (Group 2). The study found that Group 2 outperformed Group 1 in tasks involving syllabic and phonetic manipulation, phonetic segmentation, syllabic and phonetic addition, substitution, and combination [19]. Similarly, the third study, conducted with children aged 8 to 12 years, aimed to characterize and compare the performance of students with ADHD (Group 1) on metalinguistic, reading, and reading comprehension skills with a group of children with good academic performance (Group 2). The study found that Group 2 made fewer errors in tasks involving phonetic identification, phonetic subtraction, syllabic addition, and syllabic substitution [22].

The results of these studies can be explained by the fact that performance in metalinguistic skills requires significant concentration and attention from the child to identify syllables and phonemes, maintain and execute instructions, organize and plan responses, and retrieve information stored in working memory [22]. These cognitive processes are often impaired in children with ADHD, particularly those with predominantly inattentive symptoms, compared to control groups [16,17].

### 4.3. Significance of Cognitive Prerequisites for Reading Skills in Spanish

The cognitive precursors of reading in Spanish, including attentional difficulties and working memory deficits observed in children with ADHD, have been described in previous studies, which have highlighted that reading acquisition is a complex process that requires not only academic skills but also various cognitive processes necessary for successful reading. Among the cognitive processes recognized as fundamental for reading acquisition are decoding visual stimuli, speed of naming, vocabulary breadth, working memory capacity, attention maintenance, phonological skills, and executive processes including monitoring, interference suppression, and inference resolution [7,61].

In the present study, it was observed that, concerning the assessment of metalinguistic skills, the ADHD group tends to be more heterogeneous in their scores on tasks such as synthesis, sound counting, spelling, and word counting. This finding is consistent with the results of a study conducted in Canada with children aged 7 to 10 years, aimed at describing metalinguistic and executive control skills in three groups of children: those with ADHD, healthy children, and bilingual children. This study found variability in metalinguistic skill scores among children with ADHD, which was explained by the high variability in language skills within the sample [62]. The relationship between language development difficulties and reading problems had previously been reported in research with Spanish-speaking children in grades two through six. This study found that reading difficulties were more common among children with associated language problems [63,64].

Regarding metalinguistic skills, as shown in Table 7, children in the ADHD group had lower means than the control group in tasks such as synthesis, sound counting, and word counting. However, this difference was not observed between the two groups for the spelling task. This result is consistent with findings from a study conducted in Australia, which included children aged 8 to 12 years with ADHD and a control group. This study aimed to describe reading, mathematical, and spelling skills and found that both ADHD children and their peers without the diagnosis had lower scores in spelling, with the effect being more pronounced in the performance of the girls evaluated [65].

It is likely that children with ADHD performed poorly in the spelling task due to potential difficulties in visual integration processes, motor coordination, working memory, organization, and planning—cognitive processes that are particularly affected in children with ADHD [66].

The CNA reading domain encompasses measures of accuracy, comprehension, and reading speed for children aged 6 to 16 years, including various subtests that assess accuracy (syllables, words, non-words, sentences, reading aloud), comprehension (sentences, reading aloud, and silent reading), and speed (reading aloud and silent reading) [7,38,39,67,68]. This research employed a reading protocol with CNA tasks and found that children in the ADHD group had lower average scores compared to the control group across all assessed tasks of accuracy, comprehension, and speed (Table 6, Table 7 and Table 8). To better understand this point, children with ADHD often display structural and functional abnormalities in key brain regions involved in attention, executive function, and language processing, which are essential for proficient reading. Neuroimaging studies consistently reveal reduced gray matter volume and atypical development of the prefrontal cortex, particularly the dorsolateral prefrontal cortex (DLPFC) and anterior cingulate cortex (ACC) [69]. These areas are critical for sustaining attention, error monitoring, and cognitive control, processes that directly impact reading accuracy and comprehension. Additionally, the parietal lobe, implicated in visuospatial attention, and the cerebellum, which contributes to timing, sequencing, and motor control, also show structural and functional deficits in ADHD [70,71]. These neuroanatomical alterations impair the coordination and fluidity required for reading speed and accuracy.

From a neurophysiological standpoint, ADHD is characterized by dysregulation of dopaminergic and noradrenergic neurotransmitter systems, which play pivotal roles in maintaining attention and regulating cognitive control [71]. This neurotransmitter imbalance affects the communication between the prefrontal cortex and subcortical structures such as the basal ganglia, leading to impaired inhibitory control and attentional modulation, which are crucial for efficient reading and comprehension. Additionally, children with ADHD exhibit abnormal neural oscillations, specifically increased theta activity and reduced beta activity, which reflects a state of cognitive under-arousal [72]. These neurophysiological disruptions contribute to difficulties in sustaining attention and modulating cognitive processes during complex tasks such as reading.

The combined neuroanatomical and neurophysiological deficits in ADHD create a cascade of impairments that affect various aspects of reading performance. The reduced capacity for sustained attention, poor working memory, and difficulties in cognitive control—driven by both structural abnormalities in critical brain regions and neurotransmitter dysregulation—collectively hinder reading accuracy, comprehension, and speed. This interplay between altered brain structure and dysfunctional neural communication helps explain why children with ADHD consistently score lower across all assessed reading tasks compared to typically developing peers.

### 4.4. Performance Specifications of Children with ADHD in Reading: Accuracy, Comprehension, and Speed

In terms of accuracy estimation in the reading process, the ADHD group showed lower means for all reading tasks evaluated compared to the control group. This finding corroborates a study with Canadian adolescents aged 14 to 16 years, which assessed reading comprehension skills across three samples: ADHD, reading impairments, and a control group. This study reported lower scores in reading accuracy tasks, silent reading comprehension, and reading speed for both children with ADHD and those with reading disorders compared to controls [73].

Similarly, this study’s results are consistent with research conducted in the Netherlands with children aged 10 to 13 years with ADHD and a control group, which described impairments in lexical and sublexical route processing and reading aloud. This research identified deficits in sublexical route processing in ADHD individuals, evidenced by lower accuracy in phonological decision-making [74].

Difficulties in reading accuracy among children with ADHD may be attributed to challenges in the sublexical (indirect or auditory) route. This route facilitates the conversion of unknown words or pseudowords into sounds and subsequently into words using grapheme-phoneme conversion rules [75,76]. This route also supports reading aloud and decoding words through grapheme-to-phoneme conversion. For children with ADHD, reading may be slower and less accurate, particularly with irregular words that do not adhere to standard grapheme-phoneme conversion rules or involve ambiguous conversions [77].

For instance, a word like ‘epolitamo’ might be read as ‘epolítamo’, representing an accentuation error. Similarly, literal substitution errors such as ‘epolitano’ for ‘epolítamo’ and subvocalization errors in long pseudowords like ‘epolítamo’ and ‘craseplántico’ significantly impact accuracy and comprehension in the reading process [78].

Regarding reading comprehension, the study’s results indicate that children with ADHD performed worse in all evaluated tasks compared to the control group. This finding aligns with a study involving children in the United States aged 8 to 13 years, which assessed whether high working memory demands affected reading ability. Although both groups showed low performance under high working memory demands, children with ADHD exhibited a significant decrease in reading comprehension performance [79]. This result also corresponds with previous research indicating that children with ADHD read more slowly and have lower text comprehension compared to their peers [80].

In comparing both groups, no significant differences were observed in academic skill assessments for sentence reading tasks and silent reading comprehension (Table 8). This is consistent with findings from a study with Canadian adolescents aged 14 to 16 years, which identified subtle difficulties in reading accuracy and comprehension scores for silent reading, with scores remaining in the average range despite being low [81]. This may be due to silent reading requiring fewer attentional resources compared to reading aloud.

Regarding reading speed, this study found differences between groups, with the control group performing better, indicating slower reading in the ADHD group. This result supports the view that children with ADHD read more slowly and less accurately, even in tasks with lower working memory demands. This difficulty may be more pronounced in tasks with high cognitive load; generally, the slower decoding and text processing speed in children with ADHD is related to working memory development challenges. This conclusion is consistent with evidence linking working memory to reading comprehension and fluency [79].

The findings also align with research involving children in the United States aged 9 to 14 years with ADHD and a control group, which described executive components related to reading speed. This study found differences in response selection, with children with ADHD demonstrating slower verbal fluency processes and lower processing speed in reading compared to the control group [82].

The measures where children with ADHD showed lower performance than controls involve not only rapid performance but also precise and efficient responses. The preparation and selection of responses include executive components, potentially involving verbal and spatial working memory, which are fundamental to reading processing speed [83]. This cognitive effort supports both reading speed and accuracy in word reading [84].

Finally, the primary scientific finding from the results of this study is the confirmation of the impact of a diagnosis of ADHD on metalinguistic and reading skills, as evidenced by the lower performance compared to the control group. In this research, since samples were drawn from a broad age range of children aged 5 to 14 years, it can be indicated that difficulties manifest early in children and seem to persist throughout their schooling. This suggests that early identification of reading-related difficulties, which emerge as metalinguistic skills develop, could significantly enhance the subsequent reading performance of students with ADHD. Moreover, such early interventions could lead to more efficient outcomes.

The main clinical implication of confirming these metalinguistic and reading difficulties in children with ADHD is the pressing need for clinicians to enhance neuropsychological evaluation protocols. There should be greater emphasis on the assessment of language and reading abilities. Literature indicates that the most affected processes and the primary focus of pediatric assessments at this stage pertain to attention and executive function.

From a clinical-educational perspective, the findings underscore the importance of incorporating metacognitive intervention programs that allow children to develop a greater awareness of language components from the onset of their schooling, when they are learning reading skills. This would enable them to better understand and regulate their performance. Particularly, if children engage in metacognitive reflection regarding their own learning profiles, it establishes the necessity to understand their reading performance and how to incorporate meta-comprehension strategies to enhance their outcomes.

## 5. Future Research Directions and Implications

From the research tradition surrounding the study of ADHD, numerous studies have been conducted on the neuropsychological characterization of this disorder, given its high prevalence and its impact on the academic, familial, and social performance of affected children. Thus, neuropsychological profiles of children diagnosed with ADHD have been reported worldwide. However, it is likely that certain areas within neuropsychology are underrepresented in studies with ADHD children, particularly research focused on academic skills (such as reading, writing, and math) or studies exploring the relationship between motivational and emotional performance. Future research should aim to investigate these areas, given the potential benefits that enhancing the evaluation and intervention processes in academic skills could bring to children with ADHD during their school years.

In the present study, the effect of ADHD on metalinguistic abilities and general reading skills was demonstrated using a case–control sample. Future studies should include larger sample sizes to better differentiate the ADHD subtypes: inattentive, hyperactive-impulsive, and combined. Previous studies have reported lower performance in reading tasks, especially in lexical access, slower word processing, and poor phonemic skills, particularly for the inattentive subtype compared to the other subtypes [85]. However, despite the limited sample size in the present study, which restricted the establishment of distinct profiles for each subtype, the findings undoubtedly contribute to confirming the lower performance in metalinguistic and reading abilities in children with ADHD, in general, compared to control groups or other groups of children with different comorbidities.

## 6. Conclusions

The results of this study contribute to validating previous findings that children with ADHD generally score lower on metalinguistic and reading skill assessments compared to control groups. This study reaffirms these findings with a sample of Colombian children from a central region of the country, thus corroborating evidence from diverse global contexts.

The study also underscores a significant avenue for future research. The discussion and literature review highlight the crucial role of cognitive precursors in learning and effective reading processing. Many prior studies suggest that initial difficulties in metalinguistic skills, particularly phonological awareness, may underlie reading challenges in children with ADHD. However, there appears to be a gap in addressing how working memory difficulties might impact the development of these metalinguistic skills and subsequent reading abilities.

In children with ADHD, deficits in working memory can be largely attributed to dysregulation in key neurotransmitter systems, specifically those involving dopamine and norepinephrine. Dopamine plays a central role in the regulation of working memory by modulating synaptic plasticity and neural communication within the prefrontal cortex. It supports the ability to hold relevant information in mind while suppressing irrelevant or distracting stimuli, a function critical for both reading comprehension and semantic processing. In ADHD, reduced dopamine levels or impaired dopamine signaling in the prefrontal cortex can lead to difficulties in maintaining focus on a text, retaining critical pieces of information, and integrating meaning across sentences [86]. This impairment disrupts the coherence of semantic storage, as children with ADHD may struggle to maintain semantic representations in working memory long enough to draw meaningful inferences or comprehend complex information.

On the other hand, norepinephrine is involved in enhancing attention and vigilance, particularly under conditions requiring sustained mental effort, such as reading comprehension tasks. In the context of ADHD, insufficient norepinephrine transmission in the prefrontal cortex and other brain regions responsible for attention control further exacerbates working memory impairments [87]. Norepinephrine helps optimize the signal-to-noise ratio in neural circuits, allowing for the prioritization of relevant information. In children with ADHD, this regulatory mechanism is weakened, making it harder to filter out distractions, organize semantic content, and maintain comprehension over longer reading passages.

Future research should delve deeper into this area, investigating the role of working memory in the development of metalinguistic skills and reading among children with ADHD, as well as in control groups.

It is essential to recognize that working memory involves the temporary storage and manipulation of information necessary for various cognitive tasks [88], including the online storage and processing of verbal and visuospatial information [89].

In the context of reading, working memory is crucial for developing reading skills related to accuracy and speed, as well as for general language and reading comprehension (94). Research indicates that individuals with higher working memory capacity are better at integrating syntactic and pragmatic information during reading comprehension tasks. Consequently, working memory is linked to specific tasks involving reading comprehension and semantic storage [90,91]. Further investigation into the role of the phonological loop in reading, especially under conditions of articulatory suppression, could provide valuable insights [92].

## 7. Limitations

While the study successfully engaged families through face-to-face meetings to explain the study’s objectives and secure informed consent, this approach may introduce potential biases. First, the reliance on voluntary participation may lead to a selection bias, as families who are more engaged or interested in their children’s education and health may be more likely to agree to participate. This could result in a sample that does not fully represent the broader population of children with ADHD in the region. Additionally, the face-to-face nature of the meetings may inadvertently influence parental perceptions of the study, potentially leading to social desirability bias, where families might feel compelled to present their children or their circumstances in a more favorable light. Furthermore, those families who declined participation may have had different socio-economic or educational backgrounds, further skewing the sample’s representativeness. These biases should be acknowledged as they may limit the generalizability of the findings to the wider population of children with ADHD.

## Figures and Tables

**Table 2:** Quantitative variables.

Variable Group	Variable	Variable Abbrev.
Metalinguistic skills	Language metalinguistic skills synthesis task	LMST
	Language Metalinguistic Skills Sound Counting Task	LM-SCT
	Language Metalinguistic Skills Spelling Task	LM-ST
	Language Metalinguistic Skills Word Counting Task	LM-WCT
Reading	Academic Skills Reading Accuracy/Syllables	AS-RA/S
	Academic Skills Reading Accuracy/words	AS-RA/W
	Academic Skills Reading Accuracy/Non-Words	AS-RA/NW
	Academic Skills Sentence Reading Accuracy Measure	AS-SRAM
	Number of Words with Errors in Oral Reading	NW-EO
	Academic Skills Sentence Reading Comprehension Measure	AS-SR-CM
	Reading Comprehension of Oral Reading	RC-OR
	Inferential Response in Oral Reading Comprehension (ítem 4) Raw score	IR-ORCI4
	Silent Reading Comprehension of a Text	SRC-T
	Reading Speed	RS
	Silent Reading Speed	SRS

Note: LMST = Language metalinguistic skills synthesis task, LM-SCT = Language Metalinguistic Skills Sound Counting Task, LM-ST = Language Metalinguistic Skills Spelling Task, LM-WCT = Language Metalinguistic Skills Word Counting Task. AS-RA/S = Academic Skills Reading Accuracy/Syllables, AS-RA/W = Academic Skills Reading Accuracy/words, AS-RA/NW = Academic Skills Reading Accuracy/Non-Words, AS-SRAM = Academic Skills Sentence Reading Accuracy Measure, NW-EO = Number of Words with Errors in Oral Reading, AS-SR-CM = Academic Skills Sentence Reading Comprehension Measure, RC-OR = Reading Comprehension of Oral Reading, IR-ORCI4 = Inferential Response in Oral Reading Comprehension pd, SRC-T = Silent Reading Comprehension of a Text, RS = Reading Speed, SRS = Silent Reading Speed.

**Table 3:** Instruments (Psychometric Properties).

Instrument	Author	Year	Items	Cronbach’s Alpha
DSM-IV ADHD symptom checklist	APA	1994	18	0.71–0.92
Conners’ Parent Rating Scale (CPRS)	Pineda, Rosselli, Henao and Mejía	2000	22	0.75–0.94
Conners’ Teacher Rating Scale (CTRS)	Pineda, Rosselli, Henao and Mejía	2000	20	0.75–0.94
WISC-III	Wechsler, D	1949	13 Subtests	0.77–0.89
MINI-KID	Colón-Soto, Díaz, Soto and Santana)	2005	17 modules	0.81–0.96
Children Neuropsychological Assessment	Matute, Roselli, Ardila, and Ostrosky-Solis	2007	9 cognitive domains	0.85–0.98

**Table 4:** Task used from the Child Neuropsychological Assessment (CNA).

Metalingüistic Skills	
Subtest	Task Structure
-Phonemic synthesis	This task assesses the child’s ability to construct words by recognizing and processing individual phonemes. A total of eight words are presented, with one point awarded for each correctly identified word. The highest possible score is 8.
-Spelling	The child is asked to spell eight words. One point is awarded for each word spelled correctly, with a maximum score of 8.
-Sound Counting	The child is asked to count the sounds that make up each of the eight words. One point is awarded for each word correctly segmented, with a maximum score of 8.
-Word Counting	The child is asked to state the number of words in a sentence after it is read aloud. Eight different sentences are presented, and one point is awarded for each correct identification of the word count per sentence. The maximum score is 8.
**Reading—accuracy and comprehension**	
**Subtest**	**Task structure**
-Syllable Reading	The child is required to read eight syllables. One point is awarded for each correct response. The maximum score is 8
-Word Reading	The child is required to read eight words. One point is awarded for each word read correctly. The maximum score is 8.
-Non-word Reading	The child is required to read eight non-words. One point is awarded for each non-word read correctly. The maximum score is 8.
-Sentence Reading, Correct answers	In the comprehension assessment task, the child is required to read aloud 10 sentences that include instructions (e.g., “Point to a large airplane”). One point is awarded for each correctly read sentence with no errors. The maximum score is 10.
-Sentence Reading. Comprehension.	The child receives one point for correctly following the instruction immediately after reading the instruction sheet in the previous section. The maximum score is 10.
-Reading a text aloud	The child is required to read a text aloud and answer eight questions related to the text’s content. The assessment includes reading speed (number of words read per minute) and comprehension. The maximum score for comprehension is 8.
-Silent Reading of a Text.	The child is required to read a 92-word text silently and then answer 8 questions related to the content of the text. The assessment measures both reading speed (number of words read per minute) and comprehension. The maximum score for comprehension is 8 points.

**Table 5:** Statistics for Metalinguistic Skills Variables.

Test	LMST-Case	LMST-Contr	LM-SCT-Case	LM-SCT-Control	LM-ST-Case	LM-ST-Control	LM-WCT-Case	LM-WCT-Control
Minimum	0.0	0.0	0.0	0.0	0.0	0.0	0.0	0.0
Maximum	8.0	8.0	8.0	8.0	8.0	8.0	8.0	8.0
1° Quartile	1.0	2.0	2.0	4.0	3.0	3.0	0.0	4.0
Median	2.0	3.0	5.0	6.0	4.0	5.0	4.0	5.0
3° Quartile	4.0	5.0	7.0	7.0	6.0	6.0	6.0	7.0
Mean	2.6	3.3	4.4	5.4	4.1	4.8	3.7	4.8
Standard Deviation	2.2	2.2	2.7	2.3	2.3	1.9	2.9	2.4
Coef. Variation	0.9	0.7	0.6	0.4	0.6	0.4	0.8	0.5

Note: LMST = Language metalinguistic skills synthesis task, LM-SCT = Language Metalinguistic Skills Sound Counting Task, LM-ST = Language Metalinguistic Skills Spelling Task, LM-WCT = Language Metalinguistic Skills Word Counting Task.

**Table 6:** Mann–Whitney U test for metalinguistic skills.

Variable	*p* Value
LMST	0.018
LM-SCT	0.011
LM-ST	0.086
LM-WCT	0.009

Note: LMST = Language metalinguistic skills synthesis task, LM-SCT = Language Metalinguistic Skills Sound Counting Task, LM-ST = Language Metalinguistic Skills Spelling Task, LM-WCT = Language Metalinguistic Skills Word Counting Task.

**Table 7:** Mann–Whitney U test for Reading skills.

Variable	*p* Value
AS-RA/S	0.021
AS-RA/W	0.021
AS-RA/NW	0.005
AS-SRAM	0.004
NW-EO	<0.0001
AS-SR-CM	0.077
RC-OR	0.002
IR-ORCI4	0.038
SRC-T	0.093
RS	0.001
SRS	0.008

Note: AS-RA/S = Academic Skills Reading Accuracy/Syllables, AS-RA/W = Academic Skills Reading Accuracy/words, AS-RA/NW = Academic Skills Reading Accuracy/Non-Words, AS-SRAM = Academic Skills Sentence Reading Accuracy Measure, NW-EO = Number of Words with Errors in Oral Reading, AS-SR-CM = Academic Skills Sentence Reading Comprehension Measure, RC-OR = Reading Comprehension of Oral Reading, IR-ORCI4 = Inferential Response in Oral Reading Comprehension pd, SRC-T = Silent Reading Comprehension of a Text, RS = Reading Speed, SRS = Silent Reading Speed.

**Table 8:** *p*-values for Spearman’s correlation. Metalinguistic skills.

Variables	LMST	LM-SCT	LM-ST
Cases Group			
LM-SCT	<0.0001		
LM-ST	<0.0001	<0.0001	
LM-WCT	<0.0001	<0.0001	<0.0001
Control Group			
LM-SCT	<0.0001		
LM-ST	<0.0001	<0.0001	
LM-WCT	0.000	<0.0001	<0.0001

Nota: LMST = Language metalinguistic skills synthesis task LM-SCT = Language Metalinguistic Skills Sound Counting Task, LM-ST = Language Metalinguistic Skills Spelling Task, LM-WCT = Language Metalinguistic Skills Word Counting Task.

**Table 9:** *p*-values for Spearman’s correlation. Reading skills *.

Variables	AS-RA/S	AS-RA/W	AS-RA/NW	AS-SRAM	NW-EO	AS-SR-CM	RC-OR	IR-ORCI4	SRC-T	RS
Cases Group										
AS-RA/W	0.0000									
AS-RA/NW	0.0000	<0.0001								
AS-SRAM	0.0000	<0.0001	<0.0001							
NW-EO	0.0001	0.0001	0.1921	0.3538						
AS-SR-CM	0.0001	<0.0001	0.0005	0.0140	0.0055					
RC-OR	0.0005	<0.0001	0.0006	0.3392	<0.0001	<0.0001				
IR-ORCI4	0.0075	<0.0001	0.0123	0.3107	0.0018	0.0019	<0.0001			
SRC-T	0.0319	<0.0001	0.0275	0.2043	0.0149	0.0002	<0.0001	<0.0001		
RS	0.0000	<0.0001	0.0016	0.5528	<0.0001	<0.0001	<0.0001	<0.0001	0.0007	
SRS	0.0000	<0.0001	0.1173	0.9901	<0.0001	<0.0001	<0.0001	0.0001	0.0117	<0.0001
Control Group										
AS-RA/W	<0.0001									
AS-RA/NW	<0.0001	<0.0001								
AS-SRAM	0.002	0.000	<0.0001							
NW-EO	0.031	0.039	0.220	0.907						
AS-SR-CM	0.001	<0.0001	0.001	0.199	0.005					
RC-OR	0.366	0.155	0.499	0.531	0.008	0.000				
IR-ORCI4	0.340	0.285	0.127	0.759	0.030	0.008	<0.0001			
SRC-T	0.066	0.002	0.096	0.880	0.073	<0.0001	0.000	0.002		
RS	<0.0001	<0.0001	0.001	0.297	0.007	<0.0001	0.063	0.050	0.001	
SRS	0.002	0.019	0.007	0.246	0.024	<0.0001	0.054	0.038	0.016	<0.0001

Note: AS-RA/S = Academic Skills Reading Accuracy/Syllables, AS-RA/W = Academic Skills Reading Accuracy/words, AS-RA/NW = Academic Skills Reading Accuracy/Non-Words, AS-SRAM = Academic Skills Sentence Reading Accuracy Measure, NW-EO = Number of Words with Errors in Oral Reading, AS-SR-CM = Academic Skills Sentence Reading Comprehension Measure, RC-OR = Reading Comprehension of Oral Reading, IR-ORCI4 = Inferential Response in Oral Reading Comprehension Raw score, SRC-T= Silent Reading Comprehension of a Text, RS = Reading Speed, SRS = Silent Reading Speed. * Spearman correlation values: 0.00–0.19 = very weak, 0.20–0.39 = weak, 0.40–0.59 = moderate, 0.60–0.79 = strong, 0.80–1.00 = very strong.

## Data Availability

The data presented in this study are available on request from the corresponding author. The data are not publicly available due to ethical standards.
